# Exploring Fundamentals of Prion Biology Using Natural Yeast Prions and Mammalian PrP

**DOI:** 10.3390/v16050790

**Published:** 2024-05-16

**Authors:** Irina L. Derkatch, Susan W. Liebman

**Affiliations:** Department of Pharmacology, University of Nevada, Reno, NV 89557, USA; sliebman@unr.edu

The key postulate of the prion paradigm is that some proteins can take on unconventional conformations and pass these conformations to newly synthesized protein molecules with the same primary structure. The paradigm was initially proposed to explain the unusual properties of an infectious agent causing spongiform encephalopathies: Creutzfeldt-Jacob disease in humans and scrapie in sheep and goats. Since then, self-perpetuating protein conformations have been linked to many human diseases including Alzheimer’s, Parkinson’s, Huntington’s, type II diabetes, ALS, and even cancer. Furthermore, prions were discovered in many species, including *Saccharomyces cerevisiae*. Prions may be detrimental, just egoistic elements, or even functional components of the cell. The underlying structure for most cases of prion-based conformational transmission is amyloid, highly ordered filamentous aggregates, where protein molecules are stacked through the formation of inter-molecular beta-strands. This Special Issue is concerned with prion appearance, inheritance, and importance. It includes seven original articles and three reviews.

The review by Ma et al. “Recombinant Mammalian Prions: The ‘Correctly’ Misfolded Prion Protein Conformers” re-visits the main proof of the prion concept: the evidence that the PrP protein alone can lead to spongiform encephalopathy infection. Many labs converted PrP synthesized in, and isolated from, bacterial or insect cells into protease-resistant amyloid aggregates (PrP^Res^) which are strikingly similar to PrP^Sc^ obtained from diseased animals. Furthermore, these preparations could self-propagate and induce PrP aggregation in mammalian brains. However, usually, pure PrP^Res^ could not initiate neurodegeneration with distinct scrapie pathological features. Upon a detailed review of the few studies that were able to obtain PrP^Sc^ in vitro, the authors agree with a currently emerging concept that only a few of multiple aggregated self-perpetuating conformations of PrP are structurally equivalent to PrP^Sc^, and they are not prevalent in PrP^Res^ samples. The fraction of PrP^Sc^ conformers can be increased by either guiding the in vitro PrP aggregation process by adding non-protein cofactors, or by selecting for PrP^Sc^ while passaging PrP^Res^ in animals.

For prions naturally occurring in yeast, such as [*PSI*^+^], [*URE3*] and [*PIN*^+^], infectious material could be readily obtained in vitro from bacterially expressed Sup35, Ure2 and Rnq1 proteins, respectively. Furthermore, particular heritable conformational variants, or strains, of a prion can be obtained by changing the conditions of an in vitro reaction. However, it is still not known why and how different strains are formed in vivo. In his research article, “The Mutability of Yeast Prions”, King explores transmutation of [*PSI*^+^] prion strains using an elegant system that allows him to distinguish more than 23 [*PSI*^+^] strains. Such transmutations are seen as equivalent to evolution, but at the level of protein conformation. Indeed, the frequency of such transmutations is responsive to the levels of the Hsp104 heat shock protein, that in turn is modulated by various stresses. One of the main conclusions is that most newly forming [*PSI*^+^] strains are either not prone to transmutations or transmute to one of three stable strains that are likely situated in a local free energy minimum with high transitional barriers. Thus, findings from this study may shed light into the free energy landscape of amyloids.

Several other articles also use yeast experimental systems to explore the entire prion “life cycle”: from emergence, to establishing a stably propagating structure, to elimination. In “Implications of the Actin Cytoskeleton on the Multi-Step Process of [*PSI*^+^] Prion Formation”, Dorweiler et al. use 3D time-lapse microscopy and Markov modeling to see how the [*PSI*^+^] prion forms de novo in yeast cells and how actin networks facilitate this process. The authors conclude that small foci that appear upon the overexpression of the [*PSI*^+^] forming protein, Sup35, move randomly and independently of actin. The smaller the foci and the larger the cell, the faster the movement. After 20–30 min the foci coalesce into large peripheral aggregates that are too big to be transmitted to daughter cells. This coalescence is affected by the actin cytoskeleton, albeit probably indirectly, by changing the molecular crowding in the cytoplasm. Disruption of actin cables through mutations or pharmacological treatments leads to a higher frequency of induction of stably propagating [*PSI*^+^]s, likely due to more efficient transmission to daughter cells. This raises the intriguing possibility that the actin cytoskeleton protects daughter cells from prions and amyloid aggregates accumulating in older mothers.

The goal of the “Hsp40/JDP Requirements for the Propagation of Synthetic Yeast Prions” article from the Hines lab is to understand how the prion domain composition affects the interactions between prion aggregates and chaperones that are required for aggregate fragmentation to allow for transmission to daughter cells and stable propagation. The authors utilize a set of artificial prion constructs developed in the Ter-Avanesyan lab. In these (polyQX)_n_ constructs the prion domain of Sup35 is substituted with 78–104 aa long stretches of poly-glutamines interspersed with a single heterologous amino acid at every fifth residue. The authors focus on the role of the JPD chaperone Sis1, which is hypothesized to recognize prions and bring the remainder of the fragmentation machinery to the aggregate. While naturally occurring yeast prions depend on Sis1 for propagation, the requirements for JDP chaperones, or even their domains, vary broadly. The presented results are the first example of the JDP chaperone Sis1 requirement for the propagation of synthetic prions with extremely low complexity prion domains. The finding that this evolutionarily conserved chaperone appears to recognize a common amyloid structure, rather than acting through binding to specific elements within naturally occurring yeast prions, makes this work important for studies of disease-related amyloids.

The above article shows that amyloid aggregates can hijack a chaperone system that usually prevents the accumulation of misfolded proteins to instead drive propagation of the prion state. Since various studies uncover a plethora of proteins with aggregation-prone domains, one may ask why cells are not clogged with numerous prion aggregates? The review by Son and Wickner, “Anti-Prion Systems in *Saccharomyces cerevisiae* Turn an Avalanche of Prions into a Flurry”, argues that multiple cellular systems interfere with de novo prion formation, eliminate established prions, prevent prion transmission from mothers to daughters and between different wild yeast populations, or reduce prion cytotoxicity. Importantly, the inhibition of prion formation and prion propagation caused by these systems does not require overexpression or deficiency of system components and occurs in cells not subjected to stress or other changes in environmental conditions. The components of such systems were uncovered by identifying genomic deletions and mutations that lead to increased prion de novo appearance. Interestingly, the re-introduction of these deleted genes led to the elimination of some but not all prion variants that formed in their absence. Also, some of these factors are specific for particular prions. For example, the ribosome-associated chaperones aiding in nascent protein folding control the appearance of [*PSI*^+^] but not [*URE3*], and Btn2 and Cur1 are [*URE3*]-specific. This difference in specificity may be because [*URE3*] prions are generally detrimental while the [*PSI*^+^]-forming protein, Sup35, also forms a functional prion ([*SUP35/PUB1*]) [1] or protective liquid-like droplets [2], so its aggregation pathways require additional control.

The Li lab examines how cells control the [*SWI*^+^] prion in Du et al. “Identifying Endogenous Cellular Proteins Destabilizing the Propagation of Swi1 Prion upon Overproduction”. [*SWI*^+^] is the prion form of the yeast protein Swi1—a subunit of the chromatin-remodeling complex Swi/Snf. The presence of [*SWI*^+^] affects the transcription of approximately 15–28% of yeast genes. This is a more significant effect than the elimination of the Swi1 protein and is indicative of a gain of function upon Swi1 prionization. Among the 82 identified hits, 15 were analyzed further and were found to be [*SWI*^+^]-specific because they did not affect stability of other naturally occurring yeast prions. Bioinformatic analysis of the hits, led the authors to conclude that competition for Swi1 monomers between the [*SWI*^+^] prion and other Swi1 interactors, including the Swi/Snf complex, is a potential prion-curing mechanism. This suggests the intriguing possibility that the [*SWI*^+^] prion forms and is maintained when the activity Swi1 is not needed, but that [*SWI*^+^] is quickly lost upon changes in cellular or environmental conditions leading to the sequestration of Swi1 into its functional pathway.

The subject of the review by Naeimi and Serio, “Beyond Amyloid Fibers: Accumulation, Biological Relevance, and Regulation of Higher-Order Prion Architectures”, is critical because these architectures likely affect the propagation and biological activity of prion and amyloid aggregates. Higher order structures may involve lateral assemblies of amyloid filaments, structures held together by chaperones important for prion propagation, assemblies involving other components that determine prion cellular localization, and non-amyloid liquid-like droplets formed by proteins also forming amyloid aggregates. Beyond providing an in-depth review of yeast and mammalian data, the authors candidly ask if structures of in vitro made fibers, or observations made in live cells using various microscopic techniques, are relevant to prions in unperturbed living cells. Indeed, for [*PSI*^+^], different methods of protein purification yield either bundles of laterally associated fibrils or short single filaments and ring-like oligomers. For microscopic analyses, overexpression of the prion protein and stressful growth conditions change the appearance and localization of aggregates. This underscores the importance of a concerted effort to combine different experimental approaches to reveal the true nature of in vivo aggregates and their biological impact.

Yeast has also been useful in the study of human proteins, including TDP-43, that form amyloid or prion-like aggregates associated with neurodegenerative diseases. Genetic screens for modifiers of TDP-43 toxicity in the yeast system identified proteins, including Pbp1 and its human homolog ATXN2, that enhance TDP-43 toxicity in yeast and the risk of ALS risk humans. Now, in “TDP-43 Toxicity in Yeast Is Associated with a Reduction in Autophagy, and Deletions of *TIP41* and *PBP1* Counteract These Effects”, Park et al. show that deletion of a new yeast gene, *TIP41*, related to *PBP1*, also suppresses TDP-43 toxicity. Further, they show that expression of TDP-43 reduces autophagy in yeast and that deletions of *PBP1* and *TIP41*, which reduced TDP-43 toxicity, eliminated TDP-43′s inhibition of autophagy. This is consistent with the idea that TDP-43 toxicity is in part due to its inhibition of autophagy. The authors speculate that TORC1 could be the TDP-43 cellular target that causes toxicity and inhibits autophagy. It is known that TORC1 inhibits autophagy and this is prevented when TORC1 is sequestered into stress granules or the TOROID structure. Park et al. propose that expression of TDP-43 could promote the Pbp1 and Tip41 dependent release of TORC1 from sequestration, allowing it to turn autophagy off.

Finally, two research articles focus on the polymorphisms in the mammalian prion protein, PrP, and the shadow of prion protein, Sho. In “Amino Acid Substitution within Seven-Octapeptide Repeat Insertions in the Prion Protein Gene Associated with Short-Term Course” Chen et al. study genetic prion diseases, gPrDs, which are caused by mutations in the prion protein gene, *PRNP*, and account for 10–15% of all prion diseases. A subgroup of gPrDs is caused by insertions in the PRNP octapeptide repeat region, located between codons 51 and 91, OPRIs. Such insertions facilitate the conversion of PrP^C^ into PrP^Sc^. The authors analyze clinical and ancillary features of four 7-OPRI patients from a new family in a Chinese pedigree. The unique feature of these patients is that they carry an amino acid substitution in the 7-OPRI making PrP more hydrophilic. The key finding is that this substitution is associated with a later age of onset and faster disease progression. Previously only 14 patients from 6 families with 7-OPRI had been reported, and only the effect of the M/V polymorphism in the codon 129 had been investigated. This new work opens an avenue for further investigation of the molecular mechanisms of gPrDs.

The shadow of prion protein, Sho, is encoded by the *SPRN* gene with homology to the *PRNP* gene. Sho interacts with PrP and accelerates prion diseases. Furthermore, genetic polymorphisms in the SPRN gene are related to susceptibility to prion diseases. In “Novel Polymorphisms and Genetic Characteristics of the Shadow of Prion Protein Gene (*SPRN*) in Cats, Hosts of Feline Spongiform Encephalopathy”, Kim et al. pioneer the analysis of Sho in this rarely explored host for transmissible spongiform encephalopathy. They identified four specific amino acids in the feline Sho that are not present in other species. They also revealed three single nucleotide polymorphisms in the felines’ *SPRN* and initiated the in silico analysis of their possible effects on mRNA structure.

With this, we would like to thank all authors for their contribution to this Special Issue. We enjoyed reading their papers and learnt a lot. We also want to thank all the reviewers who helped us bring the articles to publication. We expect to continue working together to answer the key questions in prion biology. Obviously, we hope to see how progress in understanding the assembly, structural variations, and infectivity of prions will lead to disease treatment strategies and drug development. Indeed, while mad cow epidemic is over and transmission of BSE to humans has been blocked, sporadic and genetic CJD still occur and there is an ongoing discussion of the possibility of transmission to humans of the chronic wasting disease, which is common in some populations of elk and deer in US [3]. Also, a recent study provides evidence for plants being a vector of environmental transmission of prions in nature [4]. The importance of mechanisms of self-propagation of protein conformations is further underscored by the rapid expansion of the list of diseases associated with heritable protein aggregates. Considering the fundamental similarities of prion-like structures, the yeast model system, well represented in the articles included in this issue, will facilitate the goal of uncovering cellular factors and compartments that prevent the accumulation of toxic and pathologic aggregates. Another very intriguing aspect is the functional role of prions and prion-like structures. We wonder if many of the proteins with aggregation-prone domains are engaged in functional aggregation that is controlled by multiple chaperone and non-chaperone systems that are being uncovered in studies of non-functional, disease-related and synthetic prions.

## References

[1] Li X., Rayman J.B., Kandel E.R., Derkatch I.L. (2014). Functional role of Tia1/Pub1 and Sup35 prion domains: Directing protein synthesis machinery to the tubulin cytoskeleton. Mol. Cell.

[2] Franzmann T.M., Jahnel M., Pozniakovsky A., Mahamid J., Holehouse A.S., Nüske E., Richter D., Baumeister W., Grill S.W., Pappu R.V. (2018). Phase separation of a yeast prion protein promotes cellular fitness. Science.

[3] Trout J., Roberts M., Tabet M., Kotkowski E., Horn S. (2024). Two hunters from the same lodge afflicted with sporadic CJD: Is chronic wasting disease to blame?. Neurology.

[4] Carlson C.M., Thomas S., Keating M.W., Soto P., Gibbs N.M., Chang H., Wiepz J.K., Austin A.G., Schneider J.R., Morales R. (2023). Plants as vectors for environmental prion transmission. iScience.

